# Antihyperglycemic and Antihyperlipidemic Effects of Aqueous Extracts of *Lannea edulis* in Alloxan-Induced Diabetic Rats

**DOI:** 10.3389/fphar.2018.01099

**Published:** 2018-09-27

**Authors:** Michelo Banda, James Nyirenda, Kaampwe Muzandu, Gibson Sijumbila, Steward Mudenda

**Affiliations:** ^1^Department of Pharmacy, School of Health Sciences, University of Zambia, Lusaka, Zambia; ^2^Department of Chemistry, School of Natural Sciences, University of Zambia, Lusaka, Zambia; ^3^Department of Biomedical Sciences, School of Veterinary Medicine, University of Zambia, Lusaka, Zambia; ^4^School of Medicine and Health Sciences, Mulungushi University, Livingstone, Zambia

**Keywords:** antihyperglycemic, antihyperlipidemic, Lannea edulis (L.E), alloxan-induced diabetic rats, diabetes mellitus

## Abstract

*Lannea edulis* (Sond.) Engl. commonly known as wild grape is used traditionally for the treatment of diabetes. It is only found in Eastern and Southern Africa. Phytochemical screening, antihyperglycemic and antihyperlipidemic effects of aqueous extracts of *L. edulis* in alloxan induced diabetic rats were carried out. We report herein the findings of this research work. *Lannea edulis* crude aqueous extracts were obtained by hot infusion and evaporation method. Phytochemical screening was carried out and subsequently toxicity studies of the aqueous extracts were performed to establish the Lethal Dose 50 (LD_50_) in albino rats. Alloxan monohydrate was used to induce diabetes in the rats. *Lannea edulis positive control group doses of 100, 300, and 500 mg/kg were administered to 3 groups for 14 days. The positive control group was administered 5 mg/kg of glibenclamide. The negative and normal control groups were administered distilled water*. To determine fasting blood glucose, blood was drawn on days 0, 1, 3, 5, 7, and 14 while it was drawn on days 0 and 14 for the determination of lipids. Phytochemical screening revealed the presence of flavonoids, saponins, tannins, cardiac glycosides, alkaloids and steroids. *L. edulis* diabetic positive control groups showed significant (*P* < 0.05) dose dependent reductions in fasting blood glucose levels. When day 0 mean blood glucose levels were compared to day 3 mean blood glucose levels of their respective groups, the 300 mg/kg *L. edulis* group showed a 23.3% drop and the 500 mg/kg *L. edulis* group showed a 52.6% drop. The 100 mg/kg *L. edulis* diabetic positive control group showed a 25.1% drop by day 5, the day on which it showed statistical significance (*P* < 0.05) compared to the diabetic control. In addition, administration of aqueous extracts of *L. edulis* to diabetic rats for 14 days significantly decreased (*P* < 0.05) the levels of serum total cholesterol, triglycerides, Low Density Lipoprotein (LDL) and Very Low Density Lipoprotein (VLDL) whilst increasing the levels of High Density Lipoprotein (HDL), when compared to the diabetic control group. It was concluded that *L. edulis* showed significant and dose dependent antihyperglycemic and antihyperlipidemic effects thus confirming its traditional use.

## Introduction

Diabetes mellitus (DM) is a group of metabolic disorders characterized by hyperglycemia (Dipiro et al., 2008). It develops as a result of defects in insulin secretion, insulin action or both (Rang et al., 2014). There are two main types of DM: Type 1 which is characterized by an absolute deficiency of insulin as a result of autoimmune destruction of pancreatic B cells (Dipiro et al., 2008) and Type 2 is accompanied by both insulin resistance and insulin secretion. Type 2 DM patients are often obese (Rang et al., 2014). The chronic increase in blood glucose levels results in microvascular and macrovascular complications, which are the leading cause of morbidity and mortality associated with DM (Johansen et al., 2005). One such complication is hyperlipidemia, which is characterized by elevated levels of cholesterol, triglycerides and changes in lipoproteins (Rajaei et al., 2015).

According to the International Diabetes Federation-Africa, there are over 14 million people in Africa with DM, and this number is predicted to double by the year 2040 (International Diabetes Federation-Africa, 2015). In Zambia alone, 218,200 cases of DM were reported in 2015 with 8,232 diabetes related deaths (International Diabetes Federation-Africa, 2015). One reason for this death toll could be limited access to healthcare especially in remote areas. Another reason is lifestyle changes such as eating behavior (Wing et al., 2001).

It has been reported that traditional medicines are particularly useful in developing countries as most individuals have limited resources and access to therapy (Ali et al., 2006). Moreover, traditional medicines have recently gained popularity in both developing and developed countries due to their possession of fewer side effects and their natural origin (Modak et al., 2007). Several plant derived active principles such as flavonoids, tannins, alkaloids, glycosides, and saponins have been found to possess anti-hyperglycemic and anti-hyperlipidemic activity (Grover et al., 2002; Ojiako et al., 2013; Mannan et al., 2014).

*Lannea edulis* (Sond.) Engl. of the Anacardiaceae family is a perennial dwarf shrub that grows from 3 to 60 cm tall and is only found in Eastern and Southern African countries such as Kenya, Zambia, Zimbabwe and South Africa (Fern et al., 2016). In Zambia, its local names include *Muumbu* (Tonga), *Kambali* (Lala), and *Mlamba* (Nyanja) and its extracts are used to treat diarrhea (Flora of Zambia, 2018)^1^. In Zimbabwe, its root extracts are taken as a treatment for bilharzia (schistosomiasis), diarrhea (Chigora et al., 2007), and gonorrhea (Maroyi, 2011). *L. edulis’* flavonoids and tannins have been reported to have antimicrobial activity (Van Wyk and Gericke, 2009).

Herbalists and traditional healers in Venda of Limpopo province in South Africa use *L. edulis* traditionally to treat diabetes (Deutschländer et al., 2009). In Namibia, *L. edulis’* roots are used to treat angina pectoris and other heart conditions (Pennacchio et al., 2010). Activity-guided isolation of radical-scavenging compounds from the dichloromethane extract of the root bark of *L. edulis* led to isolation of two known bioactive alkylphenols, and three new dihydroalkylcyclohexenones (Queiroz et al., 2003). The role of antioxidant or radical scavenger plant extracts in providing a protective mechanism against reactive oxygen species (ROS) associated with chronic hyperglycemia and diabetic complications has been extensively reviewed by many (Tripathi and Chandra, 2010; Joseph et al., 2013).

Despite the traditional oral reports and practices pointing to antihyperglycemic and antihyperlipidemic activity of *L. edulis*, no studies have been performed to validate its use. Therefore, the purpose of the present study was to carry out phytochemical screening of *L. edulis* aqueous extracts and to determine whether *L. edulis* possesses antihyperglycemic and antihyperlipidemic properties or not.

## Materials and Methods

### Plant Identification

A whole plant of *Lannea edulis* (Sond.) Engl. (Anacardiaceae) was collected in December 2016 in Ndola, Copperbelt Province, Zambia. The plant was identified and authenticated by a qualified botanist from the University of Zambia (HZL) Herbarium housed in the School of Natural Sciences, Department of Biological Sciences, and deposited in the herbarium with a voucher specimen number/accession number 22,064. Only leaves of the plant were used in this study as traditionally, they have been used as a hot water decoction.

### Preparation of *L. edulis* Aqueous Extracts

The leaves of *L. edulis* were rinsed completely with distilled water to remove surface contaminants and then air dried in the shade for 3 days. A hundred and fifty grams of the leaves were ground using a Phillips Cucina blender to homogeneity. Five hundred milliliters of distilled hot water was added to the homogenate powder and allowed to sit for 30 min. Two more extractions were carried out with 300 ml of hot water per extraction (Gbolade, 2009). The water was then filtered using MN615 150 mm filter paper. The filtrate was evaporated to dryness on a hot plate and a sticky brown residue was obtained. The percentage yield was calculated and found to be 17.2%. The residue was then stored in air and water proof containers, and kept at 4°C. From this stock, a fresh preparation was made whenever required.

### Study Setting and Population

Healthy male albino rats *(Rattus norvegicus)* weighing between 140 and 200 g body weight, with no previous drug treatment, were acquired from the Animal House, Department of Biological Sciences, School of Natural Sciences, University of Zambia. The rats were taken to the Animal House at the School of Veterinary Medicine, University of Zambia and allowed to acclimatize for a period of 7 days. They were maintained under standard animal house conditions (temperature: 26–30°C, photoperiod: approximately 12 h natural light per day and relative humidity: 55–60%) with continuous access to pelleted food and water. Research was conducted in accordance with the internationally accepted principles for care and use of laboratory animals (National Research Council (United States) Committee, 2011).

### Data Collection Techniques and Tools

#### Phytochemical Screening

A phytochemical screening to determine the presence of saponins, tannins, flavonoids, anthraquinones, steroids, alkaloids and cardiac glycosides was performed using the procedures outlined (Sofowora, 1993; Trease and Evans, 2002).

#### Acute Oral Toxicity Study (Determination of LD_50_)

The Karber method was used to determine the LD50 (Chinedu et al., 2013). Seven groups of five albino rats each weighing 140–200 g were treated orally once, with different doses of *L. edulis* crude aqueous extracts (10, 100, 300, 2000, 5000, and 6000 mg/kg).

For signs of toxicity, the OECD guide (Co-operation and Development, 2002) was followed to determine toxicity, with the help of the Hodge and Sterner scale.

#### Anti-diabetic Activity in Alloxan Induced Diabetes Model

Alloxan monohydrate (Sigma Aldrich, Germany), was used to cause pharmacological diabetes to confirm the effectiveness of aqueous extracts in experimental diabetic conditions. Diabetes was induced in 80 male albino rats by injecting 120 mg/kg of Alloxan monohydrate intra-peritoneally dissolved in 0.9% w/v cold normal saline to overnight-fasted rats (12 h). The rats were afterward kept for the next 24 h on 10% glucose solution bottles, in their cages, to prevent hypoglycemia. After 72 h of injection, fasting blood glucose level was measured. The animals that did not develop more than 200 mg/dL glucose levels were omitted from the study. Thirty six (36) selected diabetic and non-diabetic animals were divided into six groups (*n* = 6). Group 1 served as normal control and was treated with 1 ml distilled water orally fed using a feeding tube. Group 2 had alloxan induced diabetes and received 1 ml of distilled water every morning; Group 3 had alloxan induced diabetes and was treated with glibenclamide (5 mg/kg/day) as the reference drug every morning. Groups 4, 5, and 6 had alloxan induced diabetes and were given 100, 300, and 500 mg/kg *L. edulis* doses, respectively. The animals were given the extracts once every morning by compulsory oral intubations before meals. The treatment was continued for fourteen (14) consecutive days.

#### Collection of Blood Samples for Glucose Analysis

Blood samples for glucose analysis were collected from the tail vein of the overnight fasted rats (12 h) after 3 days of administration of Alloxan (day 0) and subsequently on days 1, 3, 5, 7, and 14. The first blood drop was wiped away but the second was dropped onto a glucose strip in the Accu-Chek glucometer (Roche Diagnostics, Germany), to get a glucose reading. The tails were then rubbed with ethanol to prevent infection.

#### Biochemical Parameters

On days 0 and 14, blood samples for serum lipid levels were collected from overnight fasted rats under diethyl ether anesthesia by retro orbital plexus puncture method and were kept aside for 30 min for clotting. The serum was separated by centrifuging the sample at 2500 rounds per minute, for 10 min at 25°C. It was subsequently analyzed for TG, HDL, and TC using the Mission Cholesterol Meter (Acon Laboratories, Inc. San Diego, CA, United States).

Very Low Density Lipoprotein (VLDL) was calculated as Triglycerides (TG); TG/5, and LDL was estimated using the Friedewald formula (Friedewald et al., 1972) as follows:

LDL (mg/dL)=TC−(HDL+VLDL)

#### Quality Control

To ensure reliability of results, quality control was performed on the Mission Cholesterol Meter and Accu-Check glucometer according to instructions before each test analysis.

#### Data Processing and Analysis

All the values were expressed as mean ± Standard Error of the Mean (SEM). The results were analyzed for statistical significance using one-way ANOVA followed by Dunnett’s test. *P* < 0.05 was considered significant.

## Ethical Considerations

This study was carried out in accordance with the recommendations of the Guidelines for Care and Use of Laboratory Animals in Biomedical Research (National Research Council (United States) Committee, 2011). The protocol was approved by the University of Zambia Biomedical Research Ethics Committee.

## Results

### Qualitative Phytochemical Screening

Phytochemical screening on the *L. edulis* hot water leaf extracts for tannins, flavonoids, saponins, steroids, alkaloids and cardiac glycosides, tested positive. Anthraquinones tested negative (**Table 1**).

**Table 1:** Results of phytochemical screening.

Phytochemicals	*L. edulis*
Tannins	+
Flavonoids	+
Saponins	+
Anthraquinones	−
Steroids	+
Alkaloids	+
Cardiac glycosides	+

*Shows the phytochemical screening results of L. edulis leaf crude extract. Present phytochemicals are denoted by (+) sign, absent phytochemicals are denoted by (−) sign.*

### Acute Oral Toxicity Study

The LD_50_ of *L. edulis* was tested up to a dose of 6000 mg/kg and none of the doses caused death or caused rats to exhibit symptoms of toxicity. The LD_50_ value of *L. edulis* was therefore found to be greater than 6000 mg/kg and according to the Hodge and Sterner scale falls in the practically nontoxic range.

### Antidiabetic Effect in Alloxan Induced Diabetes Models

The LD_50_ of *L. edulis* was found to be greater than 6000 mg/kg so dose selection was done below it. Generally, the effective dose 50 (ED_50_), the dose at which a therapeutic effect can be produced in 50% of the subjects taking it, is considered 10 times less than the LD_50_ (Belhekar et al., 2013), and since one of the tested doses was 5000 mg/kg, a tenth of that dose (500 mg/kg) was chosen as the highest dose. Other doses included 100 and 300 mg/kg of *L. edulis*.

**FIGURE 1:**
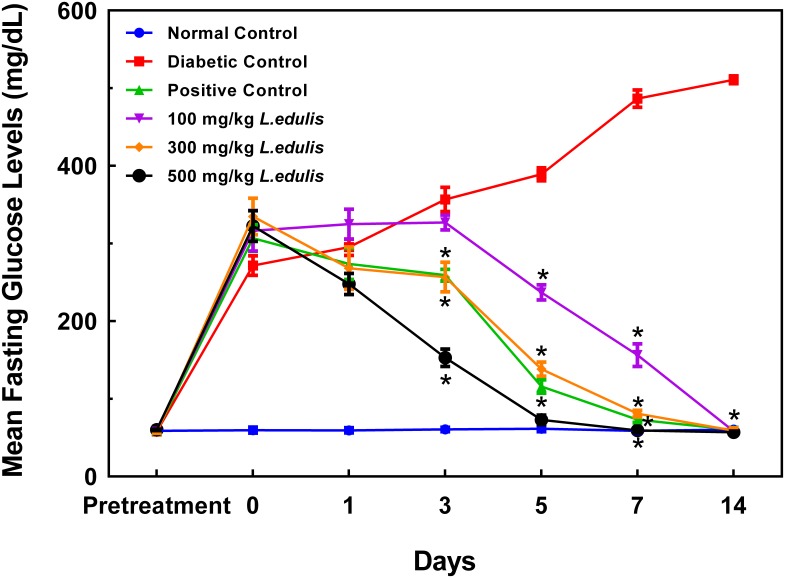
Antidiabetic effect of aqueous extracts of *L. edulis* on blood glucose levels of alloxan-induced diabetic rats for 14 days treatment. Data is expressed as Means ± SEM. ^∗^*P* < 0.05 versus diabetic control (one-way ANOVA followed by Dunnetts test).

All treatment groups had normal blood glucose levels before the induction of diabetes and were not statistically different from each other (**Figure 1**). Mean blood glucose levels after induction of diabetes (day 0 blood glucose levels) of the positive control, diabetic control, and *L. edulis* diabetic positive control groups rose to above 200 mg/dL and there were no statistically significant differences between these groups, as can be seen in the same figure. The daily dose of *L. edulis* resulted in significant reductions in blood glucose levels compared to the diabetic control from day 3. One-way ANOVA followed by Dunnetts test showed that only the positive control, 300 mg/kg and 500 mg/kg *L. edulis* diabetic positive control groups had significant differences (*P* < 0.05) in mean blood glucose levels compared to the diabetic control group from day 3. The 100 mg/kg *L. edulis* diabetic positive control group had significant difference (*P* < 0.05) compared to diabetic control group from day 5 (**Figure 1**).

Administration of the 300 mg/kg and 500 mg/kg *L. edulis* doses resulted in the attainment of normal blood glucose levels by day 5 whilst the 100 mg/kg *L. edulis* dose only achieved this by day 14 (**Figure 1**). Administration of glibenclamide 5 mg/kg (positive control group) also resulted in attainment of normal blood glucose levels by day 5. After induction of diabetes, the diabetic control group had mean blood glucose levels that continued to rise throughout the treatment period and went above 500 mg/dL by day 14 whilst the normal control group had blood glucose levels that remained in the normal range throughout the treatment period (**Figure 1**).

**FIGURE 2:**
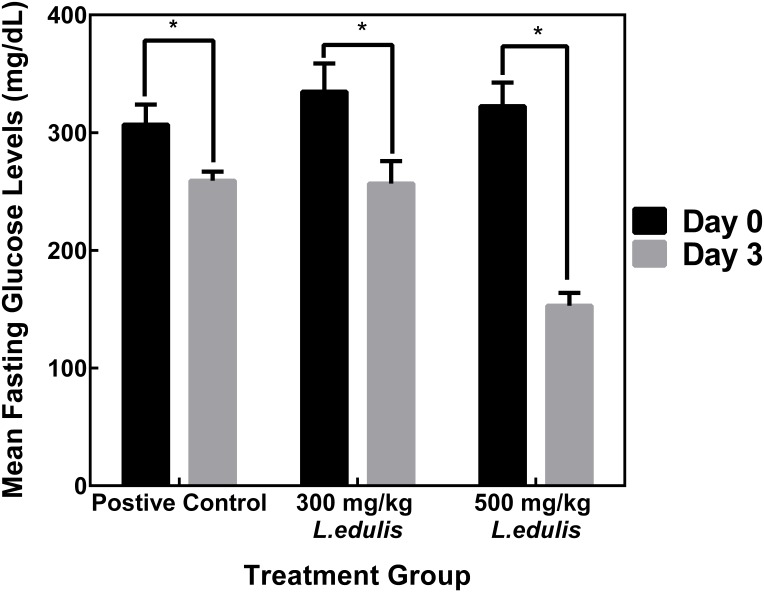
Percent reduction in mean fasting glucose levels at days 0 and 3, respectively. Administration of 300 mg/kg and 500 mg/kg *L. edulis* extracts, respectively, resulted in a significant reduction in blood sugar levels. Data is expressed as Means ± SEM. ^∗^*P* < 0.05 versus diabetic control (one-way ANOVA followed by Dunnetts test).

Comparison of day 3 mean blood glucose levels with their respective day 0 mean blood glucose levels showed a 15.5% drop in the positive control group, 23.3% drop in the 300 mg/kg *L. edulis* diabetic positive control group and a 52.6% drop in the 500 mg/kg *L. edulis* diabetic positive control group (**Figure 2**).

The 100 mg/kg *L. edulis* group showed a 25.1% drop when day 5 mean blood glucose levels (237.0 mg/dL ± 9.7) were compared to day 0 mean blood glucose levels (316.5 mg/dL ± 26.2).

Measurement of serum lipids (TC, TG, HDL, VLDL, LDL) levels on day 0 showed that there were no statistically significant differences between groups. Administration of glibenclamide and aqueous extracts of *L. edulis* to diabetic rats for 14 days showed dose dependent reductions (*P* < 0.05) in the levels of serum TC, TG, LDL, and VLDL whilst increasing the levels of HDL (*P* < 0.05) in a dose dependent manner compared to the diabetic control (**Table 2**).

**Table 2:** Results of administration of *L. edulis* on lipid levels.

Parameter	Treatment	Pretreatment levels	
		Day 0	Day 14
TC	Normal control	106 ± 1.14	104 ± 1.24
	Diabetic control	104.83 ± 0.60	309.33 ± 2.04
	Positive control	105 ± 1.13	108.83 ± 1.02*
	100 mg/kg *L. edulis*	104.33 ± 0.80	118.83 ± 2.20*
	300 mg/kg *L. edulis*	104.67 ± 1.12	112 ± 1.21*
	500 mg/kg *L. edulis*	105.5 ± 0.85	107.83 ± 2.14*
TG	Normal control	116.4 ± 0.85	115.33 ± 0.90
	Diabetic control	115.67 ± 1.09	339.33 ± 2.74
	Positive control	116.5 ± 0.76	117 ± 1.24*
	100 mg/kg *L. edulis*	116.5 ± 0.67	133.5 ± 2.04*
	300 mg/kg *L. edulis*	115.5 ± 1.61	119.67 ± 1.54*
	500 mg/kg *L. edulis*	117.67 ± 1.23	116.33 ± 1.84*
VLDL	Normal control	23.1 ± 0.22	23.07 ± 0.24
	Diabetic control	23.13 ± 0.22	67.87 ± 0.55
	Positive control	23.3 ± 0.15	23.4 ± 0.25*
	100 mg/kg *L. edulis*	23.1 ± 0.13	26.7 ± 0.41*
	300 mg/kg *L. edulis*	23.53 ± 0.32	23.93 ± 0.31*
	500 mg/kg *L. edulis*	23.27 ± 0.24	23.27 ± 0.37*
HDL	Normal control	37 ± 1.46	38 ± 1.56
	Diabetic control	37.83 ± 1.30	17.67 ± 0.80
	Positive control	36 ± 1.36	33.5 ± 1.31*
	100 mg/kg *L. edulis*	36.17 ± 1.83	22.67 ± 1.20*
	300 mg/kg *L. edulis*	36.17 ± 1.74	29.33 ± 1.52*
	500 mg/kg *L. edulis*	38 ± 1.32	40 ± 1.39*
LDL	Normal control	43.8 ± 1.55	43.7 ± 1.66
	Diabetic control	43.87 ± 1.23	223.8 ± 1.60
	Positive control	45.7 ± 1.50	51.43 ± 1.69*
	100 mg/kg *L. edulis*	44.87 ± 1.84	69.47 ± 1.90*
	300 mg/kg *L. edulis*	45.4 ± 2.23	58.73 ± 1.80*
	500 mg/kg *L. edulis*	43.97 ± 1.84	44.57 ± 2.0*

*Shows the effect on lipid levels on administration of L.edulis crude aqueous extract. Data is expressed as Means ± SEM. ^∗^P < 0.05 versus diabetic control (one-way ANOVA followed by Dunnetts test). TC, total cholesterol; TG, triglycerides; VLDL, very low density lipoproteins; HDL, high density lipoproteins; LDL, low density lipoproteins.*

## Discussion

The first objective of this study was to determine the phytochemicals present in the leaves of *L. edulis.* Phytochemical screening revealed the presence of flavonoids, saponins, tannins, alkaloids, cardiac glycosides and steroids. Several reports have implicated phytochemicals as being responsible for anti-hyperglycemic and anti-hyperlipidemic activities (Kumar D. et al., 2011; Kumar R. et al., 2011). For instance, isoflavones, tannins and crude saponins have been found to inhibit glucose transport by inhibiting sodium glucose co-transporter-1 (S-GLUT-1) in intestinal brush border cells (Tiwari and Rao, 2002; Petchi et al., 2013).

The second objective of the present study was to determine the antihyperglycemic effect of *L. edulis* in alloxan induced diabetic rats. Alloxan induces diabetes through ROS that leads to a rapid destruction of pancreatic beta cells causing hyperglycemia (Stanely et al., 2000). Hyperglycemia in turn increases the generation of free radicals by glucose auto-oxidation (Bajaj and Khan, 2012). Results revealed that the daily doses of *L. edulis* were able to cause significant reductions (*P* < 0.05) in mean blood glucose levels compared to diabetic control in a dose dependent manner, with the 300 and 500 mg/kg *L. edulis* doses bringing blood glucose levels down to normal ranges within 5 days of administration. One possible explanation for this could be the presence of antioxidants in the plant that have been reported in a previous study (Queiroz et al., 2003). Antioxidants are protective agents that inactivate the ROS which cause cell damage (Joseph et al., 2013). Interestingly, plants which have flavonoids, alkaloids, and glycosides have antioxidant activity and are claimed to possess anti-hyperglycemic effect (Petchi et al., 2013). Joseph et al. (2013) reported that the antioxidant activity of herbal hypoglycemic plant extracts may represent a protective mechanism against ROS associated with chronic hyperglycemia and diabetic complications such as microvascular and macrovascular conditions. In addition, because the use of lower dose alloxan (120 mg/kg body weight) produces partial destruction of pancreatic beta cells, the albino rats have surviving beta cells and regeneration is possible (Aybar et al., 2001). Petchi et al., 2013) reported that flavonoids present in some plants regenerate the damaged beta cells of pancreases (Petchi et al., 2013). It is therefore possible that *L. edulis* leaf extracts may have led to the regeneration of the β-cells of the pancreas and potentiation of insulin secretion from surviving β-cells, causing an antihyperglycemic effect. It has been reported that the root extract of *L. edulis* have new compounds (5-alkyl-4,5-dihydroxy-2-cyclohexen-1-ones) known to possess antioxidant properties (Queiroz et al., 2003). Therefore, it is anticipated that similar compounds may be found in the leaf extracts to sequester ROS thereby enhancing pancreatic beta cell regeneration.

On the other hand, glibenclamide, a potent sulfonylurea used orally in small doses in the management of type 2 DM, causes stimulation of pancreatic beta cells leading to the release of insulin and causes hypoglycemia (Candasamy et al., 2014). Glibenclamide metabolites;4-trans-hydroxyglibenclamide (Ml) and 3-cis-hydroxyglibenclamide (M2) have been shown to be hypoglycemic in humans (Rydberg et al., 1994). These combined effects may have equally contributed to regeneration of beta cells in the alloxan induced diabetic rats under this study.

The third objective was to determine the antihyperlipidemic effect of aqueous extracts of *L. edulis* in alloxan induced diabetic rats. The diabetic control group was observed to have increased TC, TG, VLDL, LDL, and reduced HDL on day 14 of treatment. Diabetes-induced hyperlipidemia occurs as a result of excess mobilization of fat from the adipose tissue due to the underutilization of glucose (Akpan et al., 2012). In the diabetic state, hormone sensitive lipase has an increased ability to break down stored triacylglycerols into fatty acids resulting in a larger quantity being discharged into circulation. This subsequently promotes the conversion of excess fatty acids into phospholipids and cholesterol in the liver. The phospholipids and cholesterol coupled with the excess TG formed in the liver may be discharged into the blood in the form of lipoproteins (Rajaei et al., 2015). In addition, the diabetic state renders lipoprotein lipase inactive- an enzyme that is responsible for hydrolysing triglycerides. As a result, hypertriglyceridemia and a reduction in HDL levels are observed (Pushparaj et al., 2007). Hypercholesterolemia develops in the diabetic state because insulin has an inhibitory action on β-hydroxy-β-methylglutaryl-Coenzyme-A (HMG-CoA) reductase, a key rate-limiting enzyme responsible for the metabolism of cholesterol-rich LDL particles (Murali et al., 2002).

The *L. edulis* diabetic positive control groups had significant reductions (*P* < 0.05) in TC, TG, LDL, VLDL as compared to the diabetic control whilst HDL levels were significantly increased (*P* < 0.05). Furthermore, TC and LDL levels for all *L. edulis* diabetic positive control groups were within the normal range on the 14th day of treatment and the 500 mg/kg *L. edulis* diabetic positive control group raised HDL levels to normal range by the 14th day of treatment. One explanation for this could be that increased utilization of glucose (reflected by a drop in glucose levels) led to the inhibition of lipid peroxidation and control of lipolytic hormones. A number of plants have been reported to have anti-hyperlipidemic effects in such a manner (Adeneye et al., 2011). Other studies have demonstrated that saponins isolated from different plants produce significant anti-hyperlipidemic effects mainly by suppression of cholesterol luminal absorption and also by increasing cholesterol secretion through biliary excretion (Francis et al., 2002; Ma et al., 2002).

The common factors that lead to the development of atherosclerosis and coronary heart disease in diabetes are hypertriglyceridemia, hypercholesterolemia and elevated LDL levels (Krentz, 2003). Therefore, lowering of serum lipid levels with elevation of HDL through dietary or drug therapy seems to be associated with a decrease in the risk of cardiovascular disease and related complications (Rajaei et al., 2015). This is the first study that has demonstrated not only the antihyperglycemic but also the antihyperlipidemic effects of *L. edulis*. This makes the further study and use of *L. edulis* very important as many antihyperglycemic agents merely correct blood glucose levels without sufficient control of hyperlipidemia.

## Conclusion

On the basis of the results exhibited by this study, it can be concluded that *L. edulis* leaf aqueous extract has significant anti-hyperglycemic activity in alloxan induced diabetic rats. In addition, *L. edulis* was found to be effective in managing the diabetes associated complication hyperlipidemia.

## Author Contributions

MB contributed to the design and conducted the study, collection, analysis, interpretation of data, and wrote the manuscript. JN and KM contributed to the experimental design and critically analyzed the manuscript. GS and SM participated in the manuscript preparation. All authors read and approved the final manuscript.

## Conflict of Interest Statement

The authors declare that the research was conducted in the absence of any commercial or financial relationships that could be construed as a potential conflict of interest.

## Footnotes

1 http://www.zambiaflora.com/speciesdata/species.php?species_id=136530 [Accessed:14 Jan 2018].
